# Musculoskeletal MRI protocol

**DOI:** 10.2349/biij.6.2.e16

**Published:** 2010-04-01

**Authors:** SP Tan, A Suraya, S Sa’don, A Ruzi, M Zahiah

**Affiliations:** Radiology Department, National University Malaysia Medical Centre (UKMMC), Kuala Lumpur, Malaysia

**Keywords:** MSK MRI protocol, selected sequences, standard positioning, orientation of scan planes

## Abstract

The authors propose a musculoskeletal (MSK) magnetic resonance imaging (MRI) protocol using selected sequences for common orthopaedic indications. Selected sequences allow optimal visualisation of the indicated pathology while screening for other common conditions. The authors emphasise the need for standard positioning of the patient and standard orientation of scan planes to facilitate comparison with follow-up scans.

## INTRODUCTION

The authors propose a magnetic resonance imaging (MRI) scan protocol using selected sequences for common orthopaedic indications. Suggestions for scan parameters, planes for scan localisers, the orientation of the scan planes and the scan coverage are included. This protocol was compiled for the Avanto 1.5T MRI scanner (Siemens, Erlangen, Germany) used in the authors’ department.

## OUTLINE OF THE PROTOCOL

This protocol was tabled according to the parts of the musculoskeletal system for quick and easy reference. There are six columns with headings of ‘scan parameters’, ‘sequences’, ‘localiser’, ‘orientation of scan planes’, ‘coverage’ and ‘comments’.

Under ‘scan parameters’, the authors have suggested the radiofrequency (RF) coils to use, how to position the patient in the scanner, the centring point of the scan and the field of view (FOV) of the scan. Scan parameters will vary slightly according to the make and strength of different MRI scanners.

The authors list selected sequences under the ‘sequences’ column. Sequences are selected for optimal visualisation of the indicated pathologies while allowing for the screening of other common conditions. Extra sequences can be added after these selected sequences are reviewed. The number of sequences varies according to the indication of the scan. Some protocols have thin-slice three dimensional (3D) gradient echo (GE) sequences preferably done with isotropic voxels for multiplanar reconstruction (MPR). The interslice gap is 20%. Names of some examples of these 3D sequences have been listed where applicable. All listed T1 sequences are spin echo (SE) sequences. All the T2 weighted sequences are turbo spin echo (TSE) sequences to reduce scanning time. An interslice gap of 10% is used for SE and TSE sequences.

‘Localiser’ or ‘scanogram’ is the plane of the pilot image used to plan the orientation of the scan planes and the coverage of the scan.

Under ‘orientation of scan planes’, the authors describe how the plane of the scan should be orientated. Standard positioning of the patient together with standard orientation of scan planes will produce standard images. These standard images will facilitate comparison with follow-up scans.

Under ‘coverage’, the range of the scan to ensure adequate coverage of the anatomy of the body part imaged is described.

Under the ‘comment’ column, other sequences which may be helpful in confirming the diagnosis of the indicated pathology is suggested.

## DISCUSSION

The authors did not include tumour imaging aside from screening for metastasis in the spine and for pigmented villonodular synovitis (PVNS) of the knee. MSK infections aside from infective spondylodiscitis were also not included. In the authors' department, MRI imaging of tumour and MSK infection is individualised according to the site and size of the lesion. In cases of an MSK tumour, the sequences done often depend on the contents of tumour as well.

As with any MRI protocol, periodic revision is necessary with the advent of new technical development and knowledge.
